# Is there still a place for transcranial Doppler in patients with IABP?

**DOI:** 10.1186/s13054-020-03324-4

**Published:** 2020-10-23

**Authors:** Juliana R. Caldas, Ronney B. Panerai, Rogério Passos, Ludhmila A. Hajjar

**Affiliations:** 1 Critical Care Unit, Hospital São Rafael Rede DOR, Salvador, Brazil; 2 Universidade de Salvador- UNIFACS, Salvador, Brazil; 3 Escola Bahiana de Medicina e Saúde Pública- EBMSP, Salvador, Brazil; 4 Department of Cardiovascular Sciences, University of Leicester, Leicester, UK; 5 NIHR Leicester Biomedical Research Centre, Leicester, UK; 6 Department of Cardiopneumology, Universidade de São Paulo, São Paulo, Brazil; 7 Surgical Intensive Care, Heart Institute, University of São Paulo, Av. Dr. Enéas de Carvalho Aguiar 44, São Paulo, 05403-000 Brazil

Transcranial Doppler (TCD), a relatively inexpensive and noninvasive tool, is often used to identify emboli, stenosis, vasospasm, brain death, and cerebral autoregulation (CA) [1]. When monitoring patients with intra-aortic balloon pump (IABP), the largest study published with TCD did not show neurological complications or deterioration of CA [2], but the occurrence of abnormal reverse cerebral blood flow velocity (CBFV) was not investigated.

Diastolic flow reversal in CBFV has been described in patients with IABP [3]. This pattern implies negative CBFV in late diastole and has also been described in intracranial hypertension, brain death, and comatose patients [4]. However, the clinical significance of this dramatic alteration in the CBFV waveform pattern is still unknown.

We assessed the effect of CBFV reversal on CA, in patients with IABP, as part of a randomized clinical trial [2]. After ethical approval, we included patients submitted to elective cardiac surgery, who presented reverse CBFV and provided written informed consent.

CBFV (TCD) and intra-arterial blood pressure (BP) were performed over 5 min with the IABP operating a 1:3 ratio triggered by the electrocardiogram. Second measurement was performed for 5 min without pumping assistance soon after IABP was turned OFF.

CA was assessed by autoregulation index (ARI), with impaired CA defined as ARI < 4 [2].

Paired Student’s *t* test or Wilcoxon tests were used as appropriate.

Six of 34 patients with IABP presented CBFV reversal, aged 63.9 ± 7.9 years, EuroSCORE 5 [range 3–7], and echocardiogram LVEF 40% [35–45].

CBFV (*p* = 0.68) and BP (*p* = 0.08) did not show any differences between IABP-ON and IABP-OFF (Table 1).

**Table 1:** Cerebral hemodynamic parameters and dynamic CA with IABP ON and IABP off

Patient number			IABP ON					IABP OFF		
	ARI^#^	(mmHg)	HR (bpm)	CBFV MCA (cm/s)	ETCO_2_ (mmHg)	ARI^#^	(mmHg)	HR (bpm)	CBFV MCA (cm/s)	ETCO_2_ (mmHg)
25	0	84.9	86.6	87.9	32	1.6	81.6	84.7	88.1	32
35	1.8	77.5	81.7	33.9	33	3.9	94.2	87.0	15.8	35
65	2.3	67.3	121.2	80.4	35	5.1	63.9	123.8	85.6	35
95	2.4	60.1	100.5	89.2	29	7.9	89.7	102.5	86.9	29
96	1.6	43.1	96.2	91.4	38	3.9	81.3	90.3	95.1	38
97	5.6	65.6	113.1	40.1	32	6.1	78.2	114.4	42.4	32

*ARI* autoregulation index, *EtCO2* end- tidal CO2, *MAP* mean arterial pressure, *HR* heart rate, *CBFV* cerebral blood flow velocity, *MCA* middle cerebral artery

^#^*p* < 0.05 (Wilcoxon tests) for the comparison between IABP ON and IABP OFF

For IABP-ON, ARI was 2.0 [0.0–5.6] and 4.5 [1.6–2.6] for IABP-OFF (*p* = 0.028).

Five patients showed impaired CA with IABP-ON; however, only 3 showed impaired CA with IABP-OFF (*p* = 0.545). All patients increased the ARI on IABP-OFF, when compared with IABP-ON. None of the patients had a stroke or a diagnosis of brain injury, but two patients died and another two had delirium [2].

Our previous study found that IABP did not impair CA [2], but with CBFV reversal, we observed that CA was affected and the ARI increased in all patients when IABP was removed. This finding indicates that TCD can play an important role in the assessment of patients with IABP and we suggest a systematic approach for this purpose in Fig. 1. The main use of TCD in this context is to identify and manage the potential complications of CA impairment, to prevent the deleterious effects of changes in BP, leading to ischemia or hyperperfusion [1].

**Fig. 1:**
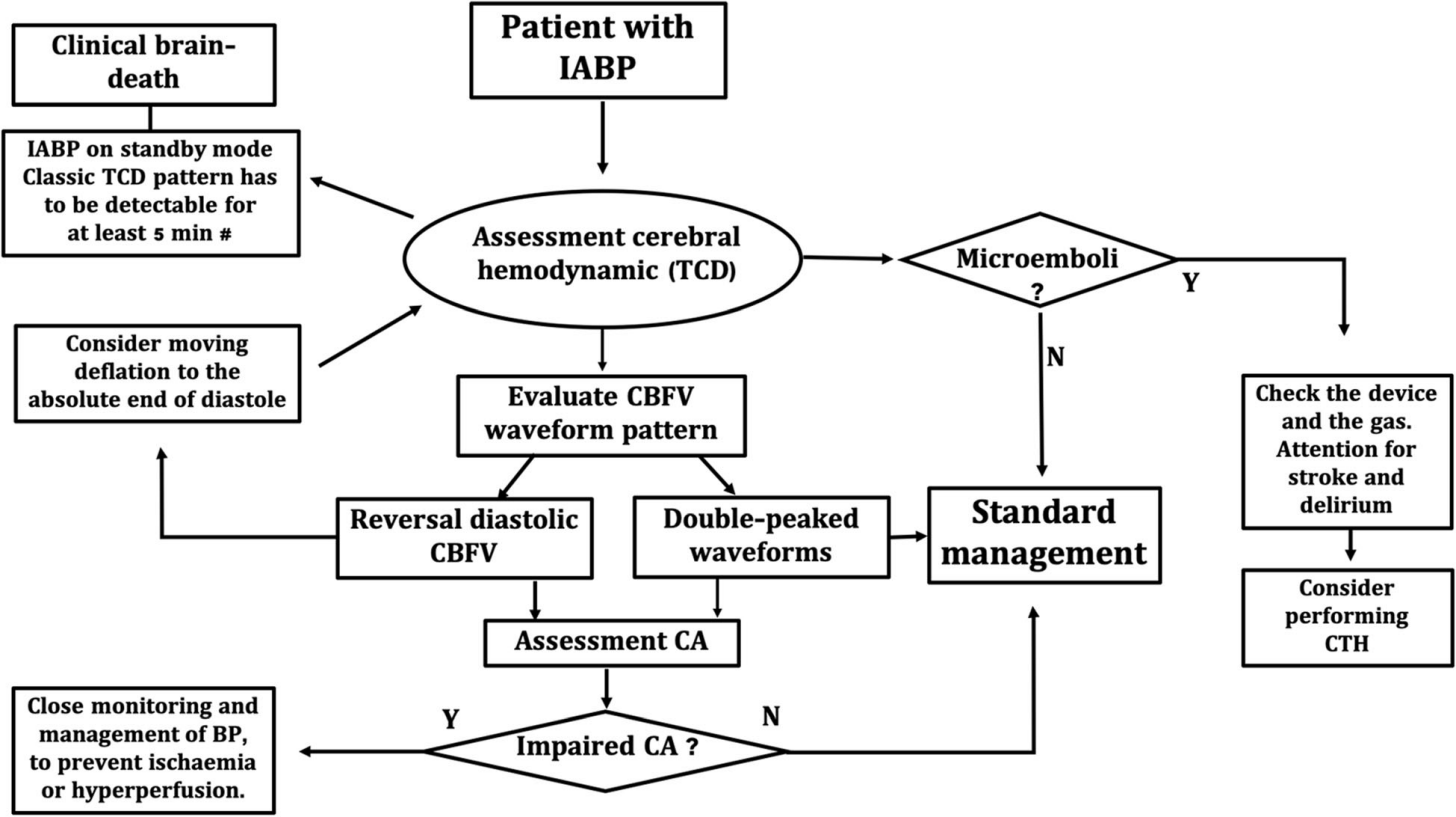
Flowchart for assessment of cerebral hemodynamics with transcranial Doppler ultrasound (TCD) in patients with intra-aortic balloon pump (IABP), proposed by the authors. CBFV, cerebral blood flow velocity; CA, cerebral autoregulation; CTH, computed tomography head. ^#^If the complementary exam is needed

CBFV reversal in IABP occurred in 17% of our cases, although it can occur in up to 35% of patients with IABP [3]. Reversal of diastolic CBFV at late diastole is probably due to rapid deflation of IABP [5], with the suggestion that CBFV reversal is iatrogenic and should be avoided. One possible approach is to optimize the balloon inflation/deflation cycle, by moving deflation to the absolute end of diastole [5]. The precise effects of CBFV reversal on cerebral hemodynamics is still unknown; its departure from normal physiological waveforms likely to be undesirable and to have a negative effect on CA.

Furthermore, negative CBFV is a TCD pattern in brain death [4]. For this reason, in IABP patients with suspected brain-death, the IABP has to be on standby to allow determination of the main source of CBFV reversal (Fig. 1).

TCD is also important to detect microemboli in IABP (Fig. 1), a cause of cerebral infarct and commonly iatrogenic in origin, often reported in cardiothoracic surgery [6]. This complication in patients with IABP, when there is a rupture with gas embolization, is rare, but potentially fatal.

Larger studies are needed in IABP patients with reverse CBFV to assess their cerebral hemodynamics and neurological outcomes.

## Data Availability

All data generated or analyzed during this study are included in this published article its additional files.
